# Influence of Seasonal Vitamin D Changes on Clinical Manifestations of Rheumatoid Arthritis and Systemic Sclerosis

**DOI:** 10.3389/fimmu.2021.683665

**Published:** 2021-06-29

**Authors:** Maurizio Cutolo, Stefano Soldano, Alberto Sulli, Vanessa Smith, Emanuele Gotelli

**Affiliations:** ^1^ Laboratory of Experimental Rheumatology and Academic Division of Clinical Rheumatology, Department of Internal Medicine and Specialties (DIMI), University of Genova, IRCCS San Martino Polyclinic, Genova, Italy; ^2^ Department of Rheumatology, Ghent University Hospital, Ghent, Belgium; ^3^ Department of Internal Medicine, Ghent University Hospital, Ghent, Belgium; ^4^ Unit for Molecular Immunology and Inflammation, Vlaams Instituut voor Biotechnologie (VIB) Inflammation Research Center (IRC), Ghent, Belgium

**Keywords:** vitamin D, rheumatoid arthritis, systemic sclerosis, circadian rhythms, connective tissue diseases

## Abstract

Vitamin D [1,25(OH)_2_D—calcitriol] is basically a steroid hormone with pleiotropic biologic effects, and its impact on the regulation of immune system may influence several clinical conditions. Calcidiol (25OHD), as precursor of calcitriol, derives, for the most part (80%), from cutaneous cholesterol (7-dehydrocholesterol) under the action of UV-B (sunlight). Consequently, serum concentrations fluctuate during the year following the circannual rhythm of sun exposition. We will update about the available evidence regarding the complex influence of seasonal vitamin D changes on two different chronic connective tissue diseases, namely rheumatoid arthritis (RA) and systemic sclerosis (SSc). Notably, RA is an emblematic model of autoimmune disease with prevalent joint inflammatory features, while SSc is mainly an autoimmune progressive pro-fibrotic disease. However, in both conditions, low serum concentrations of 25OHD are involved in the pathogenesis of the diseases, and emerging data report their impact on clinical manifestations.

## Introduction

Vitamin D plays a well-known regulatory effect on both innate and adaptive immune systems (1). In humans, vitamin D is synthesized for the most part from cholesterol (7-dehydrocholesterol) in the skin under the effects of UV-B radiation and subsequently activated into 25OHD (calcidiol) in the liver and into 1,25(OH)_2_D (calcitriol) in the kidney. For this reason, calcitriol is considered a steroid hormone (D hormone), sharing the cyclo-pentano-perhydro-phenanthrene ring structure of cholesterol with adrenal (cortisol) and gonadal (sexual) hormones (1).

25OHD deficiency is largely diffuse in general population and mainly in patients with a connective tissue disease (CTD) (2). Moreover, people face para-physiological seasonal variations of 25OHD, according to the availability of adequate solar UV-B radiations (3, 4). Hence, the aim of this mini-review is to focus on the influence of interannual variations of 25OHD serum concentrations in the clinical manifestations of two different and paradigmatic models of autoimmune diseases: an inflammatory condition, such as rheumatoid arthritis (RA) and a pro-fibrotic condition, such as systemic sclerosis (SSc).

## Vitamin D and Pathophysiology of Rheumatoid Arthritis

RA is a chronic inflammatory autoimmune disease that affects joints bilaterally with synovial hyperplasia, cartilage erosion, bone destruction, and progressive loss of function (5). Systemic organ involvement is also present due to the intense B cell response in many patients.

The pathogenesis of RA is not completely understood, but it is the consequence of a complex interaction between genetic (HLA-DRB1 and shared epitope expression), non-genetic (*e.g.*, female sex, oral and gut microbiota, diet, chronic stress) and epigenetic factors (*e.g.*, methylation and/or acetylation of the DNA induced by smoking). The final result is the generation of neo-epitopes that stimulate the innate and adaptive immune systems, with the recruitment of mononuclear cells (macrophages, T and B lymphocytes) and release of pro-inflammatory cytokines (IL-1, IL-6, IL-17, TNF-α) that invade the synovial tissue. The concomitant production of auto-antibodies (rheumatoid factor—RF and anti-citrullinated protein antibodies—ACPAs) amplifies and perpetuates the joint damage and systemic involvement (5).

1,25(OH)_2_D can act in this pathogenetic process at multiple levels. For example, estrogens are one of the major drivers of the systemic autoimmune response in course of RA. Moreover, estrogens play an intensive local B cell activity through the increased aromatase activity in human macrophages that convert androgens into estrogens in inflamed joints. 1,25(OH)_2_D is able to downregulate the expression of macrophage aromatases, reducing the production of auto-reactive B lymphocytes (6–8).

Furthermore, 1,25(OH)_2_D promotes the shift of the macrophages from the classically activated pro-inflammatory form (M1) to the alternatively activated anti-inflammatory form (M2), acting on STAT-1/TREM-1 pathway (9, 10). 1,25(OH)_2_D influences the epigenome and the transcriptome of monocytes too, and in particular the expression of vitamin D receptor (VDR) (11).

Moreover, 1,25(OH)_2_D downregulates the activity of antigen presenting cells, opposing both the release of several cytokines (IL-1, IL-2, IL-6, TNFα) and the polarization of T helper (Th) lymphocytes towards a proinflammatory response (Th1) (12).

In addition, 1,25(OH)_2_D modulates Th17 polarization in early RA patients, down-regulating IL-17A, IL-17F, TNFα, and IL-22 expression, and stimulating IL-4 that exerts anti-inflammatory effects (13). For these reasons, the association of 1,25(OH)_2_D with anti-TNFα drugs could ensure a larger spectrum of action against IL-17 and IL-22 that cooperate to synovial tissue inflammation in the early stages of RA (14).

Even if the effect of calcitriol on B cell compartment is less pronounced, low serum concentrations of 1,25(OH)_2_D have been recently associated with higher ACPA serum concentrations in early and not yet treated RA patients (15).

On the other hand, gut microbiota is an extended network of microorganisms that colonize the intestinal tract. They degrade insoluble substances and synthesize vitamin K, receiving a unique ambience for their survival in return. Dysregulation of gut microbiota has been reported as a risk factor for several autoimmune diseases, including RA (16, 17).

The reduction of *Bifidobacteria*, *Lactobacilli*, and *Prevotella copri* and the increase of *Enterobacteria* and *Staphylococcus* have been associated with a more severe RA disease activity (18). Of note, low 25OHD serum concentrations significantly correlate with increased *Escherichia coli* and reduced *Lactobacillus* and *Bifidobacterium* concentrations in the gastrointestinal tract of RA patients (19). Moreover, winter variations of 25OHD are associated with the decrease of *Actinobacteria*, *Bacteroidetes*, *Firmicutes*, and *Proteobacteria* that usually cooperate in the production of pantothenic acid. Humans need pantothenic acid in order to synthesize coenzyme A, a pleiotropic cofactor that is required for the production of cortisol. Interestingly, the supplementation of vitamin D in association with multiple vitamin B complex can restore a physiological microbiota and the normal release of cortisol, opposing a pro-inflammatory state (20).

## Seasonality of Rheumatoid Arthritis Clinical Manifestations According to Vitamin D Serum Concentrations

25OHD deficiency has been reported in several cohorts of RA patients, both at peripheral (serum) and synovial fluid levels, and it has been correlated to worse Disease Activity Score 28 (DAS28), Simplified Disease Index (SDAI), Clinical Disease Activity Index (CDAI), and Health Assessment Questionnaire (HAQ) (21–26).

**Figure 1:**
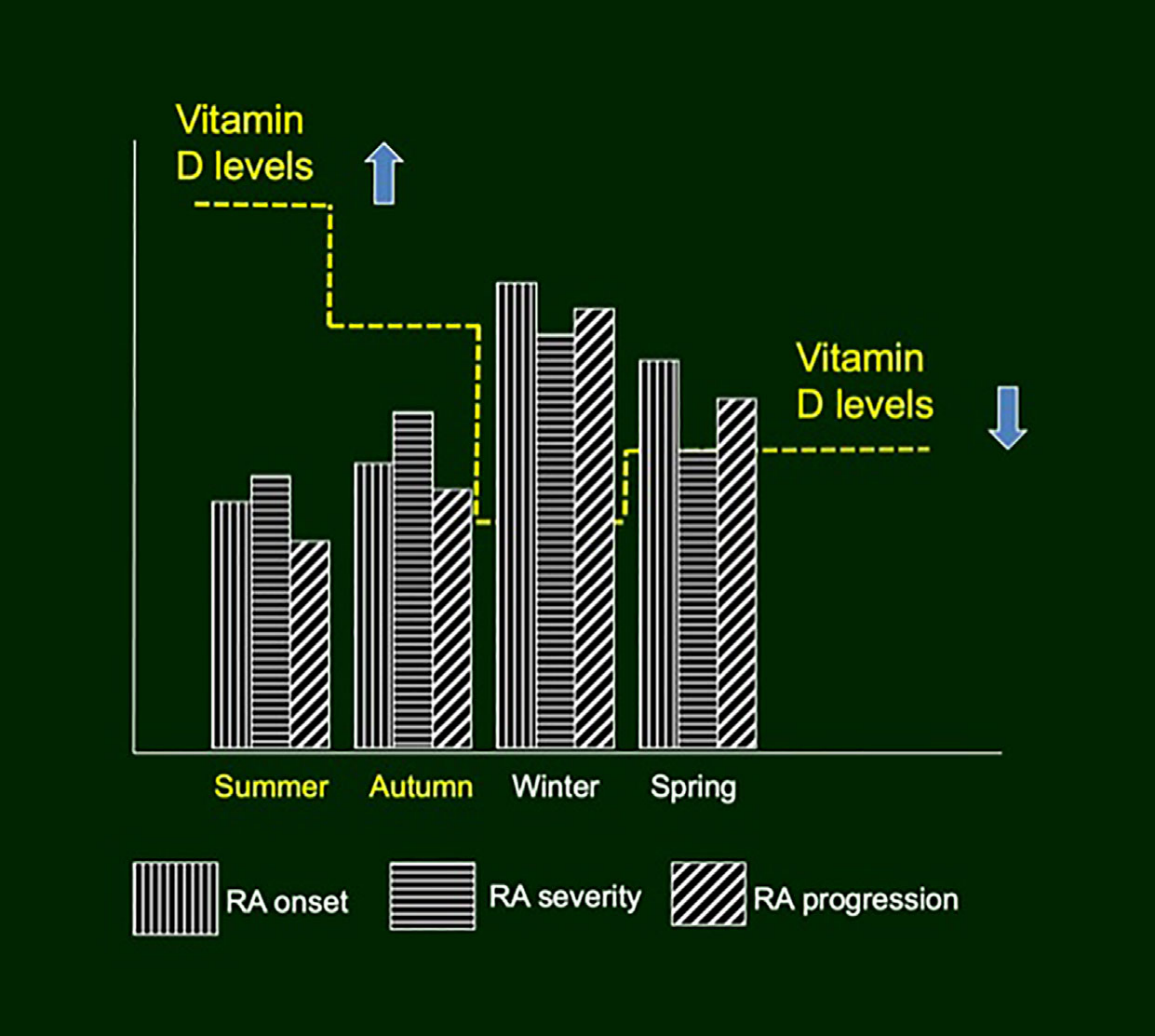
Seasonal variations of rheumatoid arthritis incidence, severity and progression, according to vitamin D serum concentrations.

Of note, subjective and objective differences between disease activity have been observed in RA patients according to the seasons. In particular, in the northern hemisphere, spring has been associated with worsening of inflammatory articular symptoms, while autumn with their improvement as evaluated by tender joint count, swollen joint count, patient-visual analog scale (VAS), HAQ, and inflammation markers (C-reactive protein—CRP, erythrocyte sedimentation rate—ESR) (**Figure 1**) (27, 28). Similarly, seasonal variations of fatigue have been described in RA patients, using both reported VAS and Bristol Rheumatoid Arthritis Fatigue Multidimensional Questionnaire, with worst results during winter (29).

Moreover, the season of RA clinical onset can predict the severity of the disease (30). In fact, in northern hemisphere onset during winter or spring (low UV and low vitamin D) rather than summer or autumn seems associated with a faster erosive radiographic progression at 6 months and with a lower probability of one-year remission (**Figure 1**) (30, 31). A large European cross-sectional study, involving 625 RA patients from 13 European countries, proposed a Patient Reported Outcome (PRO) questionnaire to self-estimate the risk for vitamin D insufficiency and deficiency-related effects on disease activity (D-PRO) (32). D-PRO consists of three sections (Symptom Risk Score, Habitus Risk Score and Global Risk Score) that well correlate with 25OHD serum concentrations, quality of life, disease activity and disability of RA patients. Consequently, the D-PRO questionnaire can help rheumatologists to identify patients that could benefit most from vitamin D supplementation (32).

## Vitamin D and Pathophysiology of Systemic Sclerosis

SSc is a multifaceted autoimmune disease characterized by a sequence of vasculopathy, dysregulated immune response, and progressive tissue/organ fibrosis. Microvascular injury is recognized as a pivotal step in the pathogenesis, almost always preceded by Raynaud’s phenomenon (RP) (33). The transition of a CTD-related RP to an irreversible microvascular damage can be easily monitored by nailfold videocapillaroscopy (NVC) that allows the visualization of microvessel specific abnormalities at high magnification (34).

As a matter of fact, persistent attacks of RP induce endothelium damage, promoting platelet aggregation and migration/activation of immune cells across the endothelium. Platelets and lymphocytes massively release pro-fibrotic mediators, such as platelet derived growth factor (PDGF), connective tissue growth factor (CTGF), and above all, transforming growth factor *β* (TGF*β*) that stimulate fibroblasts to synthesize extracellular matrix components. The final result is the fibrosis of skin and then of internal organs (35).

Calcitriol can interfere with this fibrotic process, acting on the TGF*β*-pathway by regulating Smad-dependent transcription (36). 1,25(OH)_2_D prevents the upregulation of col1a1 mRNA, and as a consequence, the synthesis of extracellular matrix proteins (*i.e.*, actin, fibronectin, collagen) and the formation of stress fibers by fibroblasts (36). Hence, calcitriol lessens the epithelial-to-mesenchymal transition of SSc-lung epithelial cells, stimulated by TGF*β* (37).

Another pathway involved in tissue fibrosis is the renin–angiotensin–aldosterone system (RAAS). Angiotensin II stimulates inflammation and fibrosis *via* angiotensin II type I receptor (AT1R) that is ubiquitously expressed (38). In the course of SSc, AT1R auto-antibodies increase the expression of IL-8 (chemotactic factor for neutrophils) and VCAM1 (adhesion molecule) (39). On the other side, angiotensin_1–7_ exerts opposite functions *via* angiotensin type II receptor (AT2R) and Mas receptor, inhibiting the MAPK/NF-kB pathway (40). SSc patients show significant lower serum concentrations of angiotensin_1–7_ with respect to healthy controls, while angiotensin II serum concentrations are normal. Consequently, the dysregulation of angiotensin_1–7_/angiotensin II ratio seems to promote SSc skin and lung fibrosis (41). In experimental animal models, 1,25(OH)_2_D can interfere also with RAAS pathway, enhancing the expression of angiotensin converting enzyme 2 that promotes the conversion of angiotensin II into angiotensin_1–7_ (42).

At last, 1,25(OH)_2_D deficiency has been associated with an over-expression of five integrins (ITGB1, ITGAV, ITGB3, ITGA4, and ITGA5) that mediate TGF*β*-induced fibrogenesis in the course of SSc (43). Coherently, two single nucleotide polymorphisms in the VDR region have been identified as susceptibility genetic alterations in the pathogenesis of SSc (44).

## Seasonality of Systemic Sclerosis Clinical Manifestations According to Vitamin D Serum Concentrations

25OHD deficiency is common in SSc patients due to intestinal malabsorption and to hyperpigmentation and fibrosis of the skin that interferes with sunlight effects (UV light) (45). In addition, in a retrospective analysis of 154 SSc patients, 25OHD deficiency has been correlated to the extent of interstitial lung disease (ILD) and to three items of Medsger’s severity scale (“peripheral vascular”, “kidney”, and “gastrointestinal” damage) (46, 47). Another study on 51 SSc patients reported the association between 25OHD deficiency and ILD, reduced diffusing capacity for carbon monoxide, cardiac diastolic dysfunction, and digital contractures (48, 49). Furthermore, two independent studies reported the link between low serum concentrations of 25OHD and the risk of digital ulcers, without finding an association with macrovascular involvement (50, 51). Coherently, Brazilian researchers described a negative correlation between vitamin D deficiency and more frequent avascular areas at NVC, as markers of more pronounced and advanced capillary damage (52).

As expected, 25OHD serum concentrations fluctuate in SSc patients during the year, according to seasons, with minimum values in wintertime in northern hemisphere (46, 53). However, to the best of our knowledge, only one study reported the influence of seasons on clinical manifestations of SSc (54). Interestingly, in a large cohort of 2,480 Thai-SSc patients, rainy season (from mid-May to mid-October) has been associated with the highest admission rate to health care system for SSc, but no prevalent causes for the admission have been reported by the Authors (54).

## Discussion

Circadian rhythms are regulated by the central nervous system and peripheral intracellular biological clocks that are essential determinants of cell synthesis, migration, and functions; they are also involved in several physiological and pathological conditions (55, 56). Large amount of evidence is nowadays available on this argument, and its relevance is certified by the 2017 Nobel Prize in Physiology or Medicine, assigned for the studies on molecular mechanisms of cell circadian clock and related circadian rhythms (57).

Circannual rhythms of incidence and relapsing of acute and chronic diseases are another fascinating, but less investigated matter of study. Even in the general healthy population of the northern hemisphere, winter season is associated with a more pronounced pro-inflammatory state, including increased serum concentrations of CRP, soluble IL-6 receptor and B-cell receptor signaling (58). It is reasonable to assume that this condition is partially due to vitamin D deficiency occurring in wintertime, as D hormone promotes an anti-inflammatory immune response (59). Similarly, a winter seasonality in the occurrence of acute viral infections, in particular of the respiratory tract (influenza virus, rhinovirus as well as SARS-CoV-2) and in the recurrence of symptoms of chronic conditions has been reported by several authors (60–64). On the contrary, both summertime and higher serum concentrations of 25(OH)D are associated with an increased number of peripheral CD4+ and CD8+ T cells with a reduced capability to produce pro-inflammatory cytokines (65).

Biological/clinical effects of seasonal 25OHD serum fluctuations seem more pronounced in inflammatory chronic diseases, as reported above in the case of RA. However, a recent large meta-analysis on patients suffering from a non-autoimmune condition such polymyalgia rheumatica and giant cell arteritis did not confirm the relevance of seasonality in the onset of the two diseases (66). Beyond common pathophysiological mechanisms, other genetic and environmental factors are involved in the pathogenesis of these inflammatory rheumatological conditions.

The aforementioned immune-modulatory effects exerted by 1,25(OH)_2_D are apparently less pronounced in non-inflammatory chronic autoimmune diseases such SSc. However, at skin level, vitamin D analogs down-regulate TGF*β*-signaling in dermal fibroblasts of both bleomycin-induced and silica-induced models of skin fibrosis (67–69).

At pulmonary level, there is an association between vitamin D deficiency and reduced lung function, evaluated by forced vital capacity (FVC), in patients with CTD-ILD (70). A retrospective study identified vitamin D serum levels lower than 20 ng/ml as a risk factor for the occurrence of CTD-ILD (71). Moreover, vitamin D has been suggested as serum biomarker of prognosis in CTD-ILD patients: in fact, lower levels are associated with a poorer prognosis, with a hazard ratio of 0.869 (72). As previously discussed, lower levels of vitamin D correlate to lower carbon monoxide diffusing capacity in the course of SSc, reinforcing the link between hormone D and lung fibrosis (48, 49).

The relationship between 25OHD deficiency and avascular areas at NVC analysis is more frequent in SSc patients with the “Late” NVC pattern of Cutolo’s classification. This observation reinforces the hypothesis of correlation between hormone D and SSc-microvascular damage (52, 73).

However, all that glitters is not gold. To date, the real impact of vitamin D supplementation on RA activity is not universally clarified, and there are contrasting reports of efficacy. By way of example, considering the two most recent meta-analyses on this topic, one found very limited beneficial effects of vitamin D supplementation on RA activity despite the high heterogeneity of the clinical studies analyzed (74). On the contrary, the second meta-analysis expressed a favorable opinion regarding the efficacy of vitamin D supplementation for RA patients: in particular, vitamin D dose ≤50,000 IU/weekly improved tender joints count only, while vitamin D dose >50.000 IU/weekly also improved VAS and DAS28 (75). The real clinical impact of vitamin D supplementation in the course of RA has yet to be elucidated by dedicated randomized clinical trials with adequate dosages and periods of assumption.

Similarly, no dedicated clinical studies are available in the course of SSc. However, as a general recommendation, a supra-physiological oral vitamin D dosage for supplementation due to the SSc-related difficulty to restore adequate 25OHD serum concentrations (at least >30 ng/ml) has been suggested (46).

At last, gender and related hormones represent an important factor to consider concerning vitamin D metabolism. In fact, physiological 17*β*-estradiol concentrations were found to decrease the expression of CYP24A1, the enzyme that usually cooperates with the 25-hydroxyvitamin D(3)-24-hydroxylase enzyme, to inactivate vitamin D (76). 17*β*-estradiol enhances the expression of VDR gene in human tissues and, consequently, women in fertile age could benefit more from vitamin D supplementation than men (76).

In conclusion, dedicated trials are desirable in the coming years to establish the optimal dosage of vitamin D supplementation to achieve a correct immune-modulation and a supportive role in the therapeutical armamentarium of at least RA and SSc.

## Author Contributions

MC, SS, and EG conceptualized the argument of the mini-review, collected the data, and wrote the manuscript. AS and VS reviewed the manuscript for important intellectual content. All authors contributed to the article and approved the submitted version.

## Conflict of Interest

The authors declare that the research was conducted in the absence of any commercial or financial relationships that could be construed as a potential conflict of interest.
